# Genetic Identity and Herbivory Drive the Invasion of a Common Aquatic Microbial Invader

**DOI:** 10.3389/fmicb.2020.01598

**Published:** 2020-07-13

**Authors:** Sarah Bolius, Karoline Morling, Claudia Wiedner, Guntram Weithoff

**Affiliations:** ^1^Department Ecology and Ecosystem Modelling, University of Potsdam, Potsdam, Germany; ^2^Department of Aquatic Environmental Engineering, Karlsruhe Institute of Technology, Karlsruhe, Germany; ^3^Leibniz Institute for Baltic Sea Research, Rostock, Germany; ^4^Berlin-Brandenburg Institute of Advanced Biodiversity Research (BBIB), Berlin, Germany

**Keywords:** alien species, genetic diversity, genotype, invasibility, cyanobacteria, consumptive resistance, phytoplankton, *Raphidiopsis*

## Abstract

Despite the increasing number of species invasions, the factors driving invasiveness are still under debate. This is particularly the case for “invisible” invasions by aquatic microbial species. Since in many cases only a few individuals or propagules enter a new habitat, their genetic variation is low and might limit their invasion success, known as the genetic bottleneck. Thus, a key question is, how genetic identity and diversity of invading species influences their invasion success and, subsequently, affect the resident community. We conducted invader-addition experiments using genetically different strains of the globally invasive, aquatic cyanobacterium *Raphidiopsis raciborskii* (formerly: *Cylindrospermopsis raciborskii*) to determine the role of invader identity and genetic diversity (strain richness) at four levels of herbivory. We tested the invasion success of solitary single strain invasions against the invader genetic diversity, which was experimentally increased up to ten strains (multi-strain populations). By using amplicon sequencing we determined the strain-specific invasion success in the multi-strain treatments and compared those with the success of these strains in the single-strain treatments. Furthermore, we tested for the invasion success under different herbivore pressures. We showed that high grazing pressure by a generalist herbivore prevented invasion, whereas a specialist herbivore enabled coexistence of consumer and invader. We found a weak effect of diversity on invasion success only under highly competitive conditions. When invasions were successful, the magnitude of this success was strain-specific and consistent among invasions performed with single-strain or multi-strain populations. A strain-specific effect was also observed on the resident phytoplankton community composition, highlighting the strong role of invader genetic identity. Our results point to a strong effect of the genetic identity on the invasion success under low predation pressure. The genetic diversity of the invader population, however, had little effect on invasion success in our study, in contrast to most previous findings. Instead, it is the interaction between the consumer abundance and type together with the strain identity of the invader that defined invasion success. This study underlines the importance of strain choice in invasion research and in ecological studies in general.

## Introduction

In recent years, globalization, along with climate and land use changes, has facilitated the spread and establishment of invasive species, which can have severe ecological impacts and are one of the biggest threats to biodiversity (Sala, 2000). Besides animals and terrestrial plants, high invasion rates are increasingly observed in (aquatic) microorganisms, supported by their high dispersal potential (Ricciardi and MacIsaac, 2010; Strayer, 2010). A prominent example for invasive aquatic microorganism are potentially toxic nostocalean cyanobacteria, an increasingly invasive order in plankton communities (Markensten et al., 2010; Sukenik et al., 2012). Although, microbes have a high potential for invasions and may have severe impacts on local communities (Sala, 2000), they are generally underrepresented in invasion research (Pyšek et al., 2008; Horňák and Corno, 2012; Strayer et al., 2017) and are rather “invisible” invaders (Litchman, 2010).

Generally, the invasiveness of a species is mainly related to three factors: (1) the characteristics of the new habitat, (2) the characteristics of the resident community, and (3) the invader itself (Lonsdale, 1999; Litchman, 2010). However, which of these factors is the main driver of the invasion process is still under debate. (1) As a prerequisite for any invasion, the abiotic conditions of the new habitat must be suitable for the invading species. If this necessity is fulfilled, (2) native communities can counteract invasions by either competitive or consumptive resistance (Alofs and Jackson, 2014). For example, effective consumers can decrease the invasibility of a community by a high consumption leading to high mortality rates of invaders (Petrie and Knapton, 1999; DeRivera et al., 2005; Juliano et al., 2010; Sperfeld et al., 2010), which has been described as the dominant type of resistance in aquatic ecosystems (Alofs and Jackson, 2014). The competitive resistance of a community relies mainly on the resource availability of the habitat, which is often low when native species-richness and/or genetic diversity is high, hampering invasions (Stachowicz et al., 1999, 2002; Fargione and Tilman, 2005; Romanuk and Kolasa, 2005). Besides the impact of the habitat and the resident community, and (3) the specific characteristics of the invader itself drives its invasion success (Crawley et al., 1999). A number of traits are linked to successful invasions, for example: short generation times, efficient resource use, tolerance to a broad range of environmental factors (Sakai et al., 2001; Litchman, 2010; Mächler and Altermatt, 2012; Mallon et al., 2015) and a high plasticity (Richards et al., 2006; Davidson et al., 2011). After establishment, invasive species directly interact with their new environment and actively modify their new habitat through, e.g., consumption or competition (Sakai et al., 2001). Thus they impair the resident community by reducing their fitness and may promote species extinctions within the community (Vilà et al., 2011). This effect on the resident community is often assumed to be species-specific (Hejda et al., 2009; Weithoff et al., 2017). Moreover, successful invasive species can change abiotic and biotic conditions to the point that it promotes the invasion of otherwise inferior invaders, leading to an invasional meltdown (Simberloff and Von Holle, 1999). Using similar reasoning, simultaneous invasions of several strains from a genetically diverse population might promote weaker strains and allows for further invasions, via changes in conditions induced by the more successful strain(s). Since the invader traits might vary among individual strains within a species, strain-specific invasions and interactions with the resident community are expected. The important role of strain identity has been shown for several biological processes, e.g., host–parasite (Barrett et al., 2009) or prey–predator interactions, for example, between *Daphnia* and *Microcystis* strains (Van Gremberghe et al., 2009; Lemaire et al., 2012). Only recently, few studies showed that strain identity has a crucial impact on the invasiveness, especially in highly variable and phenotypically plastic species such as in *Daphnia* (Engel et al., 2011) or in mammals (González-Suárez et al., 2015). Although strain identity can play a role for invasiveness, the genetic diversity of invaders is also positively associated with invasion success (Ahlroth et al., 2003; Crawford and Whitney, 2010; Forsman, 2014). Genetic diversity thereby increases the chance that one “appropriate” strain will succeed in the new habitat.

According to theory, invasive species frequently undergo a genetic bottleneck (Allendorf and Lundquist, 2003; Roman and Darling, 2007), thus decreasing genetic diversity within a species in a new habitat. However, high propagule pressure, several invasion events or invasions from different geographical regions through, e.g., human trade (Kolbe et al., 2004; Therriault et al., 2005; Roman and Darling, 2007; Lockwood et al., 2009) can counteract low genetic diversity of the invader. To date, few studies in invasion ecology have experimentally examined and followed the initial invasion process and subsequent establishment with respect to the genetic diversity and intraspecific variation of the invader (Jeschke and Strayer, 2005; Forsman, 2014; Jeschke and Heger, 2018). In addition, different strains might affect invaded communities differently, due, for example, differences in resource use efficiency.

We conducted laboratory invader-addition experiments to study the underlying mechanisms in the invasion success of the globally invasive and genetically diverse (Abreu et al., 2018) cyanobacterium *Raphidiopsis raciborskii* (formerly: *Cylindrospermopsis raciborskii*). *Raphidiopsis raciborskii* (Aguilera et al., 2018) is a bloom-forming tropical cyanobacterium, which is increasing its global distribution north- and southwards toward the temperate zones (Padisák, 1997; Antunes et al., 2015). Due to its advantageous traits, such as the capability of nitrogen-fixation, a high phosphorus affinity and storage capability and the formation of akinetes (Padisák, 1997; Wiedner et al., 2007a), it has been found to dominate also temperate phytoplankton communities (Hamilton et al., 2005; Budzyńska and Gołdyn, 2017). (i) We studied the role of consumptive and competitive resistance by varying herbivore abundances and types (specialist vs generalist). (ii) We tested the hypothesis that a high genetic (strain) diversity promotes invasions by adding different strains at varying diversity levels. Using amplicon sequencing for the multi-strain invading populations, we could determine the individual strain invasion success. In the case of successful invasions, we investigated (iii) how successful invasions affected the resident species and their community composition.

## Materials and Methods

### Model Organism

For this study, 10 genetically different *R. raciborskii* strains from Northeast German lakes were selected, which differ in their growth rates and ingestibility by the generalist rotifer *Brachionus calyciflorus* (Bolius et al., 2017). The 10 strains used here were chosen from a set of 12 strains (see Bolius et al., 2017) from established laboratory cultures. All strains were isolated between 2004 and 2010 (Wiedner et al., 2007b) and kept in batch stock culture since then.

### Experimental Design

We analyzed the invasion success of *R. raciborskii* into near-native temperate phytoplankton communities as a function of strain traits, the genetic diversity (= strain richness), and herbivory, using invader addition experiments. The resident community consisted of phyto- and zooplankton based on Sperfeld et al. (2010) and was composed of species with different functional traits such as size, growth rate, and edibility (Supplementary Tables 1, 2). To mimic complex natural conditions, competition and predation, the experimental communities consisted of phytoplankton and a herbivore. In particular in aquatic systems, consumers play a stronger role in preventing invasions than competitors (Alofs and Jackson, 2014).

**FIGURE 1:**
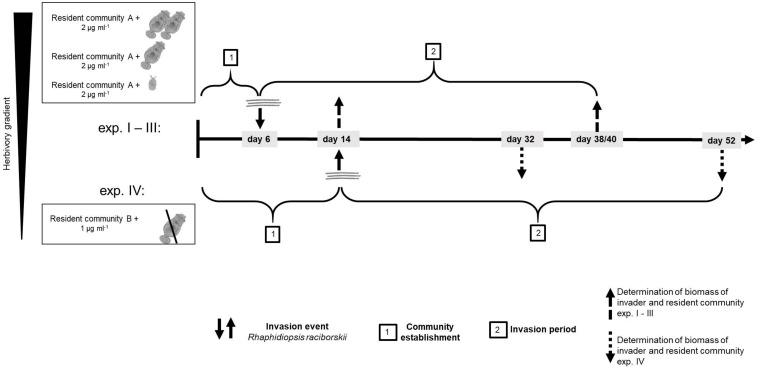
Time line of the experiments showing the four different experiments and how experiment IV differed from experiments I–III. For resident community A and B, see Supplementary Table 1.

We conducted four experiments, in two differing set-ups (Supplementary Table 1). The first experimental set-up (one experiment) was conducted with a resident community consisting of ten phytoplankton species and a high density of the generalist herbivore *B. calyciflorus* sensu stricto (Paraskevopoulou et al., 2018; strain from Fussmann et al., 2005). In this set-up, the herbivore went extinct soon after the start and before the invader addition, likely due to an increase of the large and less ingestible resident chlorophyte *Pandorina morum*. Therefore, this experiment is considered as a no-herbivory experiment. For the logic of an herbivory gradient, this experiment is named experiment IV (Figure 1 and Supplementary Table 1).

To investigate the impact of the herbivory on the invasion of *R. raciborskii* and to mimic a more natural community, a second experiment was conducted with modifications (set-up 2, three experiments, Figure 1): the composition of the resident phytoplankton community had to be changed, allowing *B. calyciflorus* to persist. However, this altered resident community enabled a high reproduction and abundance of *B. calyciflorus*. Thus, in a third experiment, we reduced the initial abundance of the herbivore, while in a fourth experiment, a smaller and more specialist consumer *Cephalodella* sp. replaced *Brachionus* (Figure 1). In addition, the sampling interval was modified to include an earlier sampling to detect short-term invasion success and its impacts, because the two (later) samplings of the first set-up resulted in similar high biomasses of the invader (see section “Results”). Thus, experiments IV (set-up 1) differed from the other experiments with respect to the species composition of the resident community, the timing of the invasion and the subsequent sampling regime (Figure 1 and Supplementary Table 1). The detailed design of the four experiments is explained and the initial parameters of the first set-up (experiment IV) are added in parentheses in the text that follows.

At the start of the experiments, the resident community was set up in 300 ml Erlenmeyer flasks (microcosms) with 150 (100) ml of modified sterile freshwater Woods Hole medium (after Nichols, 1973; 2 mM HEPES buffer, pH 8, 80 μg phosphorous L^–1^, nitrogen:phosphorous = 20:1) and was inoculated with in total 2 (1) μg ml^–1^ of a resident phytoplankton community (Supplementary Table 1) in equal parts of 0.25 (0.1) μg ml^–1^ for each species. In addition to the phytoplankton species, the resident communities included an herbivorous consumer: either the generalist rotifer *B. calyciflorus* or the smaller, more specialist rotifer *Cephalodella* sp. (Altermatt et al., 2011; Seifert et al., 2015). The four different herbivory levels were generated as follows (the experiments are numbered by decreasing herbivory): for a high level of herbivory, the initial density of *B. calyciflorus* was 1.5 animals ml^–1^ (experiment I, set up 2) and for a lower level of herbivory, 0.2 animals ml^–1^ (experiment II, set up 2). A low and more specific herbivory was generated adding one *Cephalodella* sp. ml^–1^ to the microcosms (experiment III, set up 2). Experiment IV started with 2.2 animals ml^–1^ of *B. calyciflorus*, but the herbivore declined drastically soon after the start and eventually died out (see above, first experimental set-up), resulting in no grazing pressure during the invasion period. In experiments II and III, three flasks were set-up without herbivory as no-herbivory control. Before adding the animals, food algae from stock cultures were removed by filtering the animals through gauze (for *B. calyciflorus* 50 μm and for *Cephalodella* sp. 20 μm). All phytoplankton and rotifer species were pre-cultured at 20°C, 18:6 h light-dark cycle as batch stock cultures. These conditions for temperature and light-dark cycle were chosen as they reflect the typical conditions in summer in NE Germany where the strains were isolated from. After an establishment period for the resident community (experiments I–III: 6 days; experiment IV: 14 days), the invader *R. raciborskii* was added, mimicking one single invasion event. The invader was added either as a single strain or in multi-strain populations (genetic diversity). In particular, *R. raciborskii* was added in an increasing genetic diversity gradient from one strain to a genetic mixture of up to 10 strains, with up to 15 replicates per treatment (number of replicates experiments I–III/experiment IV): one (2/3 replicates per strain), three (15/9 replicates, but different strain populations – see Supplementary Figure 1), six (15/9 replicates, but different strain populations), nine (two replicates), all 10 (three replicates), and none (as a control: three replicates without invader addition). In experiment IV the maximum genetic diversity treatment was nine strains (Supplementary Table 1). For all experiments and diversity treatments, the strains were randomly selected from the 10 strain-pool (see above and Supplementary Figure 1) and each time the invader was added with the same total biomass of 0.1 μg ml^–1^ (5% of initial phytoplankton resident community), resulting in a lower absolute biomass of each strain in the genetic mixtures (0.033 to 0.01 μg ml^–1^ per strain).

The addition of the invader comprises different forms of replicates: for the single strain treatments and nine respectively 10 strains, the same strain/strains was/were added three times, for the treatments with three and six strains all replicates consisted of different strain combinations (technical replicate, Supplementary Figure 1). In addition to the cyanobacteria, 1 ml of their filtrate (mixture of all strains; 0.8 μm, cellulose nitrate filters, Sartorius, Göttingen, Germany) was added to each flask to exclude a possible effect of potential strain-specific cyanobacteria-associated heterotrophic bacteria on the invasion success.

All experiments were run at 20°C at a light-dark cycle of 16:8 h and a light intensity of 130 μmol photons m^–1^ s^–1^ (measured in water with a spherical light sensor, Li-Cor, SQSA 0107, WALZ Mess- und Regeltechnik, Effeltrich, Germany). To reduce sedimentation, all flasks were shaken gently 15 min h^–1^. Every second day, 20% of the experimental volume was exchanged with fresh medium (dilution rate = 0.16 day^–1^). A total of 5 ml were used for regular chlorophyll-*a* fluorescence measurement (Fluorometer TD 700, Turner Designs, Sunnyvale, CA, United States) as an estimate of total phytoplankton density, the remaining volume was fixed with Lugol’s iodine, for subsequent analysis. Halfway through the experiments, the flasks were exchanged to avoid phytoplankton wall growth. The experiments lasted for 40/40/38/52 days (experiments I, II, III, and IV), respectively. Experiment III was two days shorter due to laboratory logistics.

### Non-genetic Sample Analyses

The phytoplankton species composition, including the invader *R. raciborskii*, was determined twice: either 8 days after the invasion (day 14; experiments I–III) for short-term changes, or 22 days after invasion for intermediate term changes (day 36; experiment IV) and in all experiments 34/34/32/38 days after invasion (experimental day 40/40/38/52) for long-term changes. Rotifer abundance was determined for every sampling day. All zoo- and phytoplankton samples were analyzed using inverted light microscopy (AxioVision A1, Carl Zeiss, Jena, Germany; AxiovertS100, Carl Zeiss; ALTRA20, Olympus Soft Imaging Solutions GmbH, Münster, Germany), except for *Synechococcus elongatus*, which was quantified after acridine orange staining on black 0.2 μm membrane filters (Whatman Nuclepore, GE Healthcare, Maidstone, United Kingdom) using epifluorescence microscopy (Axioscop2, Carl Zeiss). Phytoplankton cell dimensions were measured (30–50 per species) with a computer-aided image system (Cell B, Olympus Soft Imaging Solutions GmbH) and the biovolume was calculated by multiplying the abundances with the cell volume assuming appropriate geometrical shapes (Hillebrand et al., 1999). Since the strains of *R. raciborskii* cannot be unambiguously morphologically distinguished, biomass data were not separated by strain.

As a measurement of a resource use trait, at the end of the experiments I–III, particulate carbon (C), nitrogen (N), and phosphorous (P) were measured and the molar C:N:P ratios of the phytoplankton communities calculated. For particulate C and N, samples were vacuum filtered on GF/C filters (precombusted for 4 h at 450°C; Whatman), dried and measured using an elementary analyzer (EA 3000, EuroVector S.p.A, Milan, Italy; software: Callidus Software, EuroVector S.p.A). Particulate P was measured after filtration on 0.45 μm membrane filters (PALL Corporation, Port Washington, NY, United States). Determination followed the blue molybdate method by Murphy and Riley (1962), after digestion with H_2_SO_4_, K_2_S_2_O_8_, and heat (121°C; 1 h autoclaving) at a photometer (880 nm; UV Mini 1240 UV-VIS spectrophotometer, Shimadzu, Kyoto, Japan). For in-depth analysis, including strain-specific invasion success in genetic mixtures and strain-specific effects on the resident community experiment III was analyzed further. In this experiment the invader and the herbivore persistently coexisted.

### Genetic Analyses

In experiment III, the composition of the genetic mixtures was analyzed, to identify the individual strain-specific invasion success in the populations.

#### Amplicon Amplifying and Sequencing

Prior to DNA extraction, the phytoplankton samples of each mixture at the day of the invasion event and at the end (see above) were centrifuged and stored at −20°C. DNA was extracted using Metagenomic DNA Isolation Kit for Water (Epicentre, Madison, WI, United States). The *R. raciborskii* strains used here did not differ in common marker genes, e.g., *cbcBA* or *ITS* (unpublished data) and therefore, we designed primers for unique genetic markers/amplicons to distinguish each strain from all others (see Supplementary Material “Primer Design”). Following DNA extraction, from all mixtures, strain-specific markers (GenBank MT531416–MT531536) were amplified – using unique primers (Supplementary Table 3). After determining the DNA concentration (NanoDrop 2000, Thermo Fisher Scientific, Waltham, MA, United States), the amplified products of each mixture were pooled and purified (innuPREP PCRpure Kit, Analytik Jena, Jena, Germany). All PCRs were run in 20 μl aqueous reaction volume containing 1× PCR buffer, 200 μM dNTPs, 0.5 M primer, 0.02 U/μl Phusion high-fidelity DNA polymerase (Thermo Fisher Scientific) and approximately 10 ng of DNA. The initial denaturation was at 98°C for 30 s, followed by 30 cycles of denaturation at 98°C for 10 s, annealing at 64°C for 30 s, extension at 72°C for 20 s, and a final extension at 72°C for 10 min. To determine the relative share of each strain next generation sequencing was used. Libraries were constructed from amplicons and sequenced on an Illumina NextSeq to generate 150 bp paired-end reads. SeqPrep (John, 2011) was used for adapter trimming, quality filtering (q 30), and read merging. This resulted in 1,700 to 908,000 merged read pairs per sample (median 533,000).

To determine which strains were present in each of the 69 samples (33 mixtures of the invasion event and 36 of the end), the merged read pairs from each sample were compared to each of the determined unique sequences that make up the expected PCR products (see Supplementary Material “Primer Design”). To exclude the possibility of cross-contamination of PCR primers/products, this included the comparison of amplicons with sequences of PCR products for which no primer pair had been used in the given experiment. For this analysis, we used the merged read pairs as queries and the determined unique PCR products as database for BLAST searches (Altschul et al., 1990). Only alignments with 100% sequence identity and the exact length of an expected PCR product sequence were scored.

The number of each PCR product was counted as “abundance” and the relative shares of each strain were determined. As the invader was added into the experiment by biomass, we used the relative data of the invasion event (day 6) as the “DNA amount base line” and the relative gain or loss of each strain in the genetic mixtures until the end was calculated from that as a measurement for strain-specific invasion success. When the strains MEL07 and Peter07_149, as well as ZIE05 and ZIE11 were in one mixture, their individual share could not be determined due to the absence of an appropriate discriminant sequence (see Supplementary Material “Primer Design”). Therefore, in genetic mixtures with both strains present, the data were combined (named as “MEL07 and Peter07_149”; “ZIE05 and ZIE11”). One genetic mixture with three strains (sample 47) was excluded from further analysis, as the included strain 27F11 was not detected in the start sample.

### Statistical Analysis

To test for an effect of strain richness on the invasion success we calculated the invasion yield as the natural logarithm of the ratio between the observed yield of the invader and the expected yield estimated from single-strain treatments. For each diversity level, the mean and the 95% confidence interval (CI) were calculated. A positive effect of strain richness on the invasion success occurred, when the mean was positive and the 95% CI did not include zero. We included diversity levels of three and six strains. To compare two data-sets, a *t*-test was applied. Either an ANOVA for normal distributed data, or Kruskal–Wallis test as a non-parametric test was performed for comparing more than two groups using the data analysis software SigmaPlot (13.0). When significant differences were found, a pairwise multiple comparison test was applied (Tukey *Post hoc* test). Normality of data was tested using the Shapiro–Wilk test. Linear regression or Spearman rank correlation was applied to test for relationships or correlations between variables. To compare the strain-specific invasion differences, we calculated the coefficient of variation (CV) as the standard deviation divided by the mean, calculated from the relative share in the whole community. To evaluate a potential strain-specific effect on the resident communities, we performed a PERMANOVA (in R, R Development Core Team 2018, RStudio 1.0.136, function “adonis” of the package “vegan” with Bray–Curtis distance; with 10,000 permutations) for the one strain treatments. For the PERMANOVA, the composition of the resident communities (in biomass; without invader) was used. The composition of the resident communities was ordinated using a principal component analysis (PCA; in R, package “stats”) to illustrate the similarity of the resident community compositions for experiment III, day 38.

## Results

We examined the strain-specific invasion success of the cyanobacterium *R. raciborskii* at different levels of genetic diversity and four levels of herbivory.

### Impact of the Herbivore Pressure on the Invasion Successes

**FIGURE 2:**
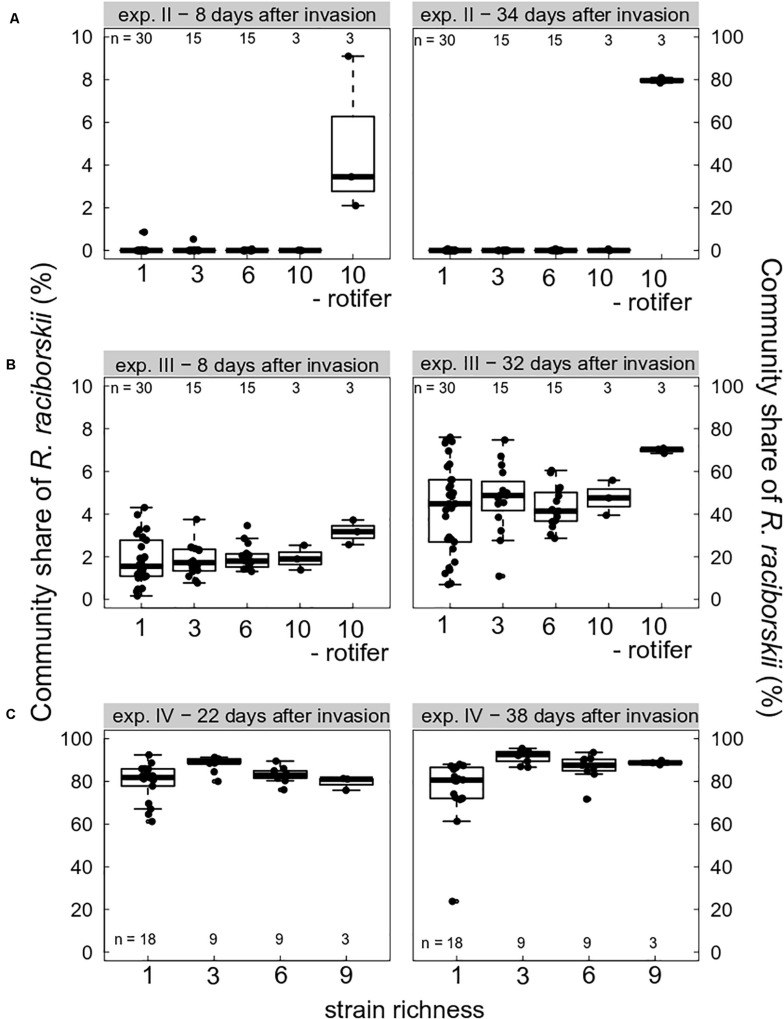
Boxplot (with data points) showing invasion success of *Raphidiopsis raciborskii*, measured as the biomass percent contribution of the resident community composition and as a function of strain richness (genetic diversity) at days 8, 22, 32/34, and 38 after invasion in experiments **(A)** II (intermediate grazing pressure, *Brachionus calyciflorus*), **(B)** III (low grazing pressure, *Cephalodella* sp.), and **(C)** IV (no grazing pressure, extinction of *B. calyciflorus*). Microcosms without consumers are indicated with “- rotifer.” Note different scales on the *y*-axes. *n* indicates the total number of microcosms. For one-strain treatments there were three **(A,B)** or two **(C)** replicates per strain; for the diversity levels three and six there were *n* mixtures with random strains and for levels nine and 10, there was only one combination possible replicated three times.

Grazing pressure strongly drove the invasion success of *R. raciborskii*. On the one hand, when grazing pressure was high due to high abundance of the consumer *B. calyciflorus*, the invasion of *R. raciborskii* completely failed (experiment I, not shown). At the first sampling on day 14 (8 days after the invasion), no *R. raciborskii* was detected using the applied method. On the other hand, without herbivory, due to consumer extinction (experiment IV, Figure 2C), and in the intended rotifer-free treatments (in experiments II and III, Figures 2A,B), *R. raciborskii* successfully invaded all plankton communities, reaching on average more than 80% of total community biomass (Figure 2). When *B. calyciflorus* abundance was at an intermediate level on the invasion date (experiment II), an invasion of the invader was possible (Figure 2A). However, the successful invasion was mainly the result of an “either or” pattern: when the consumer eventually went extinct shortly after the invader addition, successful invasions at a low level were observed in six out of 63 cases (day 40), and when the consumer reached high abundances, the invader failed or remained at very low levels (negative significant Spearman rank correlation between abundance of invader and consumer, rho = 0.650, significant at *p* < 0.0001). Only with *Cephalodella* sp. as the consumer, was coexistence possible with high abundances of both consumer and invader: 8 days after invasion, *R. raciborskii* contributed to 1.9 ± 0.9% of the phytoplankton community (Figure 2B). Until day 38 (32 days after invasion), *R. raciborskii* strongly increased its relative biomass share up to 72% (mean ± SD, 44 ± 17%), dominating most of the communities (67% of all microcosms; Figure 2B). The abundance of *Cephalodella* sp. was not correlated with the invasion success of *R. raciborskii* (linear regression, experiment III day 14: *R*^2^ = 0.021, *p* = 0.235; day 38: *R*^2^ = 0.001, *p* = 0.774).

### Strain-Specific Invasion Success

**FIGURE 3:**
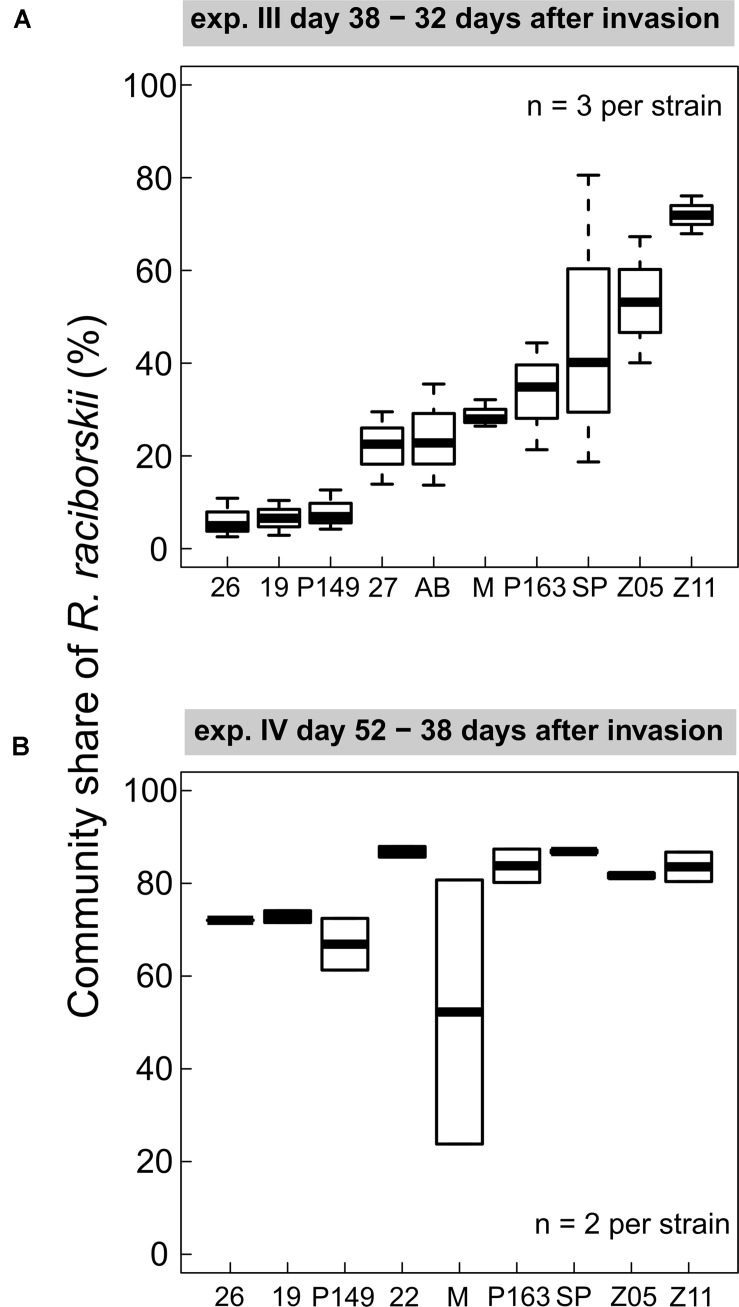
Individual strain invasion success of *Raphidiopsis raciborskii* (boxplots; in % of total community share) in experiments with **(A)** *Cephalodella* sp. (experiment III), 32 days after invasion, *n* = 3; **(B)** experiment IV with extinct herbivore, 38 days after invasion, *n* = 2. Abbreviations of the strains: 26, 26D9; 19, 19F6; P149, Peter07_149; 22, 22F8; 27, 27F11; AB, AB2008/71; M, MEL07; P163, Peter07_163; SP, SP08_4; Z5, ZIE05; Z11, ZIE11.

We found consistent strain-specific differences in the invasion success in experiments III and IV (Figure 3). Some strains were highly invasive, while others invaded the communities with lower densities, consistently between both experiments (positive Spearman rank correlation between strain invasion success of experiments III and IV, rho = 0.762, significant at *p* = 0.028), although on different absolute biomass and community composition. In experiment IV the overall invasion success was higher than in experiment III (Figure 2). In experiment III, strain-specific differences were high, expressed as a CV of 0.48, calculated from the relative share in the whole community. In experiment IV, the strains were on a more similar level (CV = 0.11), except for one outlier (MEL07, Figure 3B).

### Low Impact of the Genetic Diversity

**FIGURE 4:**
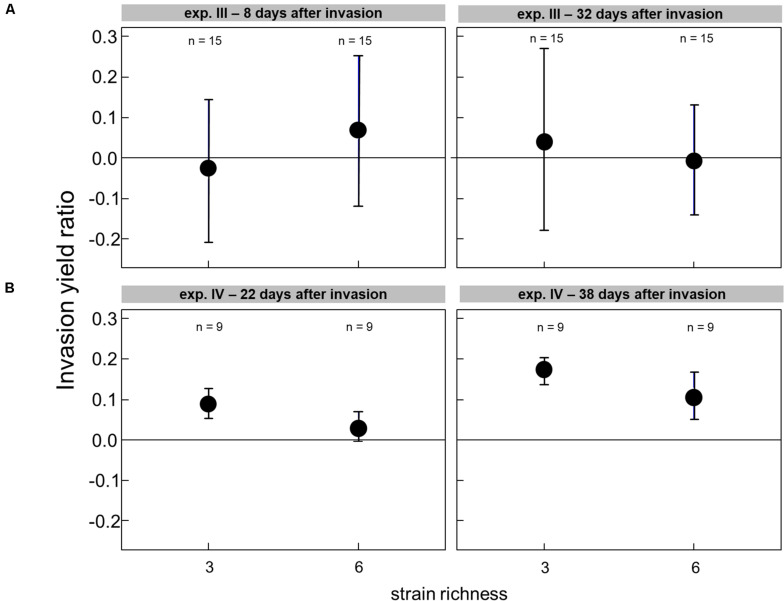
The invasion yield of *Raphidiopsis raciborskii* in experiments with *Cephalodella* sp. (experiment III) **(A)** and extinction of *Brachionus calyciflorus* (experiment IV) **(B)** for the diversity levels of three and six strains.

The effect of the genetic diversity on the invasion success could only be analyzed for experiments III and IV, with successful invasions. In the experiment with a low grazing pressure imposed by *Cephalodella* sp., the invasion success of *R. raciborskii* was not affected by the genetic diversity (experiment III, the invasion yield was not significantly different from zero; Figure 4A). The average *R. raciborskii* community share was very similar in all treatments, but the variance decreased with increasing diversity on both sampling days (see Figure 2). With no grazing pressure (experiment IV), the relative invasion success of *R. raciborskii* was only positively affected by the genetic diversity of the three-strain treatments (day 36 and 52) and for the six-strain treatments at day 52 (Figure 4B); comparison of invasion success: day 36 and 52, ANOVA, *p* < 0.001, Tukey *Post hoc* test, *p* < 0.006).

### Temporal Dynamics of the Community

Over the course of the experiments, the resident community composition changed and was partly affected by the invasion of *R. raciborskii*. When *R. raciborskii* invaded the community without consumers (experiment IV), *Chlorella vulgaris*, *Monoraphidium minutum*, *Navicula pelliculosa*, and *Oocystis marsonii* had significant different biomasses among invader diversity treatments (ANOVA or Kruskal–Wallis test, *p* < 0.009): *C. vulgaris* increased, whereas the other species decreased.

In all four experiments, some species eventually went extinct: *Aphanizomen gracile* (experiment IV), *Stephanodiscus hantzschii* (experiments I–III), *Peridinium* sp. (experiment I–III), and *Planktothrix aghardii* (experiment I–III). Among the persisting species, the cell size (cell volume for algae; filament width for filamentous cyanobacteria) changed from the experiment start to the end: *R. raciborskii* and *P. aghardii* decreased their filaments width (*R. raciborskii*: 1.8 to 0.9 μm; *t*-test, *p* = 0.018; *P. aghardii*: 5.8 to 4.8 μm; *t*-test, *p* = 0.329), the chlorophytes *Acutodesmus obliquus* (67 to 105 μm^3^; *t*-test, *p* = 0.123) and *Chlamydomonas reinhardtii* (235 to 416 μm^3^; *t*-test, *p* = 0.014) increased their cell size (see Supplementary Table 2).

### Strain-Specific Changes of the Resident Composition

**FIGURE 5:**
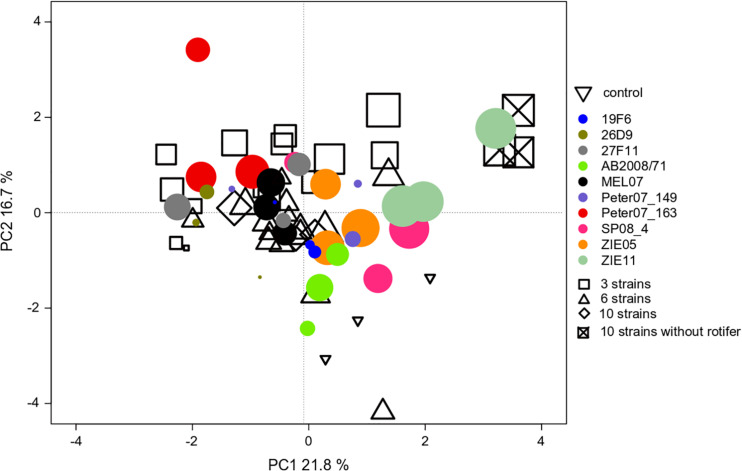
PCA ordination of the individual microcosm community compositions in experiment III with *Cephalodella* sp. on day 38 (69 microcosms = points in total). The size of the filled circles reflects the biomass contribution of *Raphidiopsis raciborskii* to the whole phytoplankton community (but not implemented in the ordination). The colors of the filled circles indicate different strains, open symbols indicate the strain richness treatments (3, 6, and 10 strains of genetic mixtures). The open triangles represent the uninvaded control (no invasion of *R. raciborskii*).

We found an effect of the strain identity on the community compositions at the end of experiment III (Table 1), where *R. raciborskii* successfully invaded. This resulted in a higher similarity of the resident communities invaded by the same strain (one strain treatments replicates) than for those invaded by different strains, as observed in the PCA of the community composition data in experiment III (in absolute biomass) excluding the invader, day 38 (Figure 5). In addition, the uninvaded control was consistently different from all other invaded treatments (with one exception), indicating a directed change in the resident community after invasion.

**TABLE 1:** Results of PERMANOVA on the effect of strain identity on resident community composition for experiments III and IV.

	***R*^2^**	***p***
**Experiment III**		
Day 14	0.3333	0.3777
Day 38	0.5729	**0.0018**
**Experiment IV**		
Day 36	0.5964	0.1332
Day 52	0.6309	**0.0396**

*Bold number indicates significance.*

**FIGURE 6:**
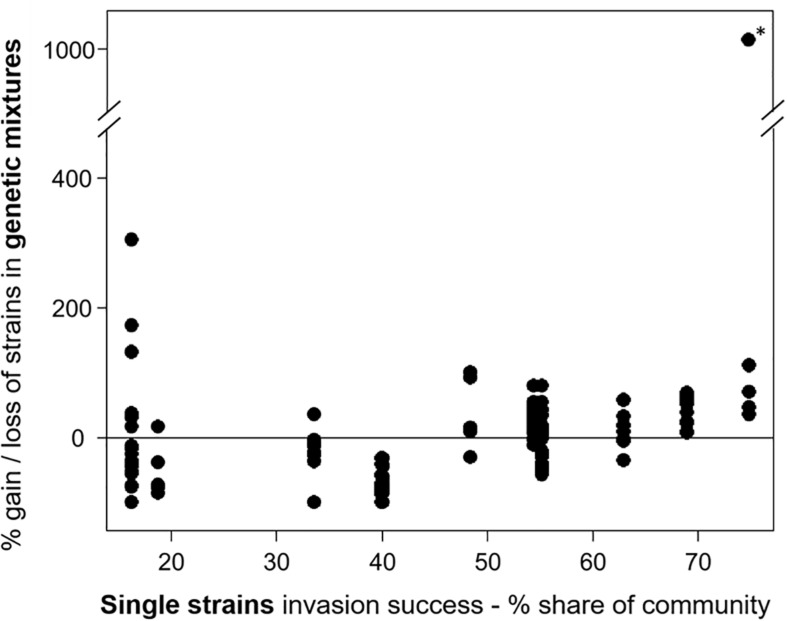
The invasion success of the *Raphidiopsis raciborskii* strains as their gain or loss in the genetic mixtures (start–end, measurement of invasion success) versus single strains. Asterisk marks the extreme outlier.

For experiment III, the individual strain share in the invading populations at the time point of the invasion event and at the end of the experiment was determined. The comparison of the relative community share of each strain between start and end revealed that some strains consistently gained and others lost in their relative share (Supplementary Figure 2). In eight mixtures (6 × 3 strains, 2 × 6 strains), one strain was dominant at the end, with a share of more than 66%, including the strains MEL07, Peter07_149, ZIE05, and ZIE11 (Supplementary Figure 3). Apart from a few exceptions, the success of specific strains in mixtures agreed with the success in single strain experiments (Figure 3) with a positive Spearman rank correlation (rho = 0.504, significant at *p* < 0.01; Figure 6). One extreme outlier (see Figure 6 marked with an asterisk) was result of a low relative share of the strain ZIE11 at the day of the invasion event. Nevertheless, the positive Spearman rank correlation remains significant (rho = 0.496, significant at *p* < 0.01) even when that outlier is included.

### Nutrients

At the end of experiment III all microcosms with *R. raciborskii* exceeded the N-concentration of the medium (51 μmol), by, on average, 78 μmol and the biomass of *R. raciborskii* was positively correlated with the particulate N (linear regression: *R*^2^ = 0.633, *p* < 0.001), indicating N_2_-fixation of *R. raciborskii*. Both, the molar C:N (8:1 to 17:1) and C:P (393:1 to 1,223:1) ratios were considerably higher than the Redfield ratio of 6.6:1 (C:N) 106:1 (C:P), indicating substantial nutrient limitation. In contrast, the controls without the N-fixing *R. raciborskii* had a nearly two-fold higher C:N ratio, likely because of the lack of N-fixation.

## Discussion

### High Grazing Pressure Can Prevent Aquatic Invasions

Our results have demonstrated an invasion resistance of resident communities driven by consumption, which is consistent with previous studies (Sperfeld et al., 2010; Alofs and Jackson, 2014; Weithoff et al., 2017). The observed consumptive resistance was somewhat surprising given the highly variable susceptibility to herbivory of the strains we used (Bolius et al., 2017). The comparison of different top-down pressures and types (high vs low and generalist vs specialist) clearly showed that one suitable consumer in the system hampers invasions, as here, most often the generalist (Maron and Vilà, 2001). The high grazing pressure by the generalist rotifer *B. calyciflorus* utterly prevented the invasion, even though *R. raciborskii* is a poor single food source for zooplankton (de Bernardi and Giussani, 1990; Panosso and Lürling, 2010). However, a mixed diet with highly edible green algae (Soares et al., 2010) and a low density of *R. raciborskii* at the invasion event (Panosso and Lürling, 2010; Soares et al., 2010; Sperfeld et al., 2010; Rangel et al., 2016) allowed for persistent herbivory. In contrast, the smaller, specialist consumer *Cephalodella* sp. could not prevent the invasion and *R. raciborskii* invaded successfully the communities (experiment III), with no obvious impact of the consumer. Due to its smaller size and likely specific grazing on *C. reinhardtii*, *Cephalodella* sp. potentially fed much less on *R. raciborskii* than the large, generalist *B. calyciflorus*.

### Strain-Specific Invasion Success Can Be the Result of a Higher Resource-Use Efficiency

The overall invasion success of *R. raciborskii* varied among strains (Figure 3). This strain-specific invasion effect was illustrated by the consistency between the two independent experiments III and IV, even though the resident communities in the two experiments differed in their phytoplankton species composition and the herbivore type and pressure. Such a consistent behavior among the strain’s invasion success was also found between their invasion success as single strains and within genetic mixtures (experiment III): the highly successful single strains also reached a higher gain in the relative share in the invading populations. This high variability in the invasion success is a reflection of their high trait variability. In a previous study, Bolius et al. (2017) found for these strains a high variability in morphology, resource use efficiently and susceptibility to herbivory (see above).

Our data indicate that the most relevant trait for a successful invasion was the intracellular N-content resulting from N-fixation (see Supplementary Table 4) and their differences among the strains. The strain-specific N-fixation correlated positively with the strains’ C:N ratio under nutrient-depleted conditions (Bolius et al., 2017), suggesting a more efficient resource use efficiency and high biomass production of some strains. Another possible supporting mechanism might have been the decrease in filament width over the time course of the experiment. Thinner filaments result in a higher surface to volume ratio, allowing for a better nutrient uptake per unit volume, increasing their competitive abilities (Friebele et al., 1978).

### Weak Effect of the Genetic Diversity on the Invasion

In most studies, a positive effect of the genetic diversity on the invasion of species is documented (see the meta-analysis in Forsman, 2014). In our study, we found a strain-specific invasion success, and only a weak effect of genetic diversity (strain richness). This resulted in a relatively constant average invasion success at all diversity levels (experiments III and IV; Figures 2B,C). However, a higher genetic diversity lowered the probability of a very low invasion success, because the presence of one or more successful strains is very likely and, thus, at a strain richness of 10 strains all invading populations had an intermediate invasion success (32 days after invasion, Figure 2B).

In general, positive effects of diversity during invasion are expected, because a high diversity results in a greater chance of including a highly invasive strain, known as portfolio or sampling effect. A low variability among invading strains would result in a low sampling effect due to a limited chance of including a strong competitor. Forsman (2014) showed in a meta-analysis (including 18 studies) a larger effect of the genetic diversity under natural conditions in the field/wild compared to laboratory experiments. He explained this effect by a higher environmental complexity. In our experiments, complexity was reflected by generating a multi-species resident community and by adding a second trophic level to mimic natural conditions. However, we used microcosms with a reduced spatial complexity typically favoring direct interactions over indirect ones (Sarnelle, 1997). Thus, the result of our study might be different from the dominant outcome in the meta-analysis from Forsman (2014).

Alternatively, an increase or a shift in resource use efficiency might occur with increasing strain richness (complementary effect; Fargione and Tilman, 2005). In our study, we found highly invasive strains in both single-strain treatments and in multi-strain populations. To maintain the total invader inoculum constant across all treatments, the individual invader/strain inoculum was ten times higher in the single-strain treatments than in the 10-strain mixtures (0.1 vs 0.01 μg ml^–1^). As a consequence, competitively successful strains started from a lower population density in mixtures and were accompanied by competitively inferior strains. Since maximal population growth rates under comparable laboratory conditions are at a low to intermediate level ranging from 0.2 to 0.4 d^–1^, it is likely that superior strains might outcompete others in the long run. At purely competitive conditions (= no consumers), Weis et al. (2007) found that the effect of species richness on biomass production can vary over time and depends on the growth rate-biomass production relationship of the competing species and also on the experimental design: additive versus substitutive (as in the present study). However, in our experiments, in experiment III in particular, the strain-specific effect is most likely much larger than the diversity effect.

In experiment IV, with a different resident community (Supplementary Table 1), the variability among the single-strain-specific invasion success was much lower than in experiment III (Figure 2). Since the consumers went extinct, no nutrient regeneration through feeding took place. Under these competitive conditions, a slightly higher observed invasion success was found than expected from single-strain experiments pointing to a potential complementarity effect (Figure 4).

The establishment of one strong species potentially can lead to an alteration of the resident community allowing otherwise less competitive species also to invade, known as invasional meltdown (Simberloff and Von Holle, 1999), and often observed on the species-level (Jeschke et al., 2012). Transferring this mechanism to our system, we found that weak invaders identified from the single-strain experiments also lost in their relative share over the course of the experiment (Supplementary Figures 2, 3), thus no invasional meltdown was observed in our study. A high invasion success was correlated with high intra-cellular nitrogen content and thus associated with nitrogen-fixation. Another factor for the strain-specific success might be allelopathy of *R. raciborskii* (Figueredo et al., 2007; Antunes et al., 2012). However, for the strains used in this study, allelopathy was not examined.

### Individual Strains Differentially Affect Resident Communities

We found a strain-specific invasion success and additionally a strain-specific differentiation of the resident community compositions (Figure 5 and Table 1), which was independent of the quantity of the invasion success (e.g., Hejda et al., 2009). A similar result was found in a study on the cord-grass *Spartina* sp., showing strain-specific responses of the neighboring plants (Zerebecki et al., 2017). Since the strains used in our study have a high variability in their traits (Bolius et al., 2017), differential effects on the resident community and their individual species could be expected. The invading strains differed, e.g., in their resource use efficiency, which might have differentially affected the resident communities. In general, this interaction between the specific invading strain and the resident species might then vary in a community context. One important consequence would be that different results are obtained from different experiments with different strains regarding the invasion process and success of species, especially in plastic species like *R. raciborskii*. This might lead to contrasting results on the invasion potential of species.

## Conclusion

To conclude, our experiments revealed that (i) a high level of herbivory hampered invasion pointing to consumptive resistance; (ii) a strong strain-specific impact on the invasion success of the invader, with only a minor effect of genetic diversity of the invading population; and (iii) a strain-specific impact on resident community composition. For *R. raciborskii*, but also for many other invasive species, genotype-specific pre-adaptation might play an important role for the establishment in a new environment. Since individual strains affect resident communities differently, their long-term persistence might also be different due differences in the competitive arena. In the long run, genetic bottlenecks might increase in importance, if the environment changes and genetic diversity is a key for long-term persistence. Strain-specificity in the invasion process and its potential consequences for long-term persistence under changing environments is a promising research avenue for future studies.

## Data Availability Statement

The datasets for this study can be found at https://doi.org/10.5061/dryad.d2547d811.

## Author Contributions

GW, CW, and SB conceived the study. SB and KM performed the experiments. SB, KM, and GW analyzed the data. SB and GW wrote the manuscript supported by comments from KM and CW. All authors contributed to the article and approved the submitted version.

## Conflict of Interest

The authors declare that the research was conducted in the absence of any commercial or financial relationships that could be construed as a potential conflict of interest.
