# Supplementary material for: Genetic Identity and Herbivory Drive the Invasion of a Common Aquatic Microbial Invader
Source: Front Microbiol. 2020 Jul 13;11:1598. doi: 10.3389/fmicb.2020.01598 (PMC7370804; doi:10.3389/fmicb.2020.01598)
Supplement: Supplementary file 1 [file Data_Sheet_1.pdf]

Supporting Information

Supplementary Table 1 Set-up of the 4 experiments (exp.) with respect to the herbivory, levels of genetic diversity, total invasion time and composition of the resident communities. Experiment (exp.) IV was the first set-up and then modified to maintain a predator population.
* indicate resident species present in all experiments. SAG: Culture Collection of Algae, Göttingen, Germany; NIVA: Norwegian Institute for Water Research, Oslo, Norway

exp.	Herbivory	Genetic diversity levels (replicates per strain/mixture)	Invader addition and community determination (experimental day)	Resident community (strain)
I	High, unselective<br>*Brachionus calyciflorus*	1 (3)<br>3 (15)<br>6 (15)<br>10 (3)<br>10 without herbivore (3)	Invasion event: day 6<br>Invader biomass determined 8 (day 14) and 34 (day 40) days after invasion	*Acutodesmus obliquus (*SAG 276-3a)*<br>*Chlamydomonas reinhardtii* (SAG 11-32b)<br>*Cryptomonas sp.* (26-80)*<br>*Monoraphidium minutum* (243-1)*<br>*Peridinium* sp. (SAG 2017)<br>*Planktothrix aghardii* (NIVA-CYA 34)<br>*Stephanodiscus hantzschii* (SAG 1020-1a)<br>*Synechococcus elongates* (SAG 89.79)
II	Low, unselective<br>*Brachionus calyciflorus*	1 (3)<br>3 (15)<br>6 (15)<br>10 (3)<br>10 without herbivore (3)	Invasion event: day 6<br>Invader biomass determined 8 (day 14) and 34 (day 40) days after invasion	*Acutodesmus obliquus (*SAG 276-3a)*<br>*Chlamydomonas reinhardtii* (SAG 11-32b)<br>*Cryptomonas sp.* (26-80)*<br>*Monoraphidium minutum* (243-1)*<br>*Peridinium* sp. (SAG 2017)<br>*Planktothrix aghardii* (NIVA-CYA 34)<br>*Stephanodiscus hantzschii* (SAG 1020-1a)<br>*Synechococcus elongates* (SAG 89.79)
III	High, selective<br>*Cephalodella* sp.	1 (3)<br>3 (15)<br>6 (15)<br>10 (3)<br>10 without herbivore (3)	Invasion event: day 6<br>Invader biomass determined 8 (day 14) and 32 (day 38) days after invasion<br>here: analysis of mixtures at day 38	*Acutodesmus obliquus (*SAG 276-3a)*<br>*Chlamydomonas reinhardtii* (SAG 11-32b)<br>*Cryptomonas sp.* (26-80)*<br>*Monoraphidium minutum* (243-1)*<br>*Peridinium* sp. (SAG 2017)<br>*Planktothrix aghardii* (NIVA-CYA 34)<br>*Stephanodiscus hantzschii* (SAG 1020-1a)<br>*Synechococcus elongates* (SAG 89.79)

Resident Community A

IV	None (extinction)<br>*Brachionus calyciflorus*	1 (2)<br>3 (9)<br>6 (9)<br>9 (2)	Invasion event: day 14<br>Invader biomass determined 22 (day 32) and 38 (day 52) days after invasion	*Acutodesmus obliquus* (SAG 276-3a)*<br>*Aphanizomenon gracile* (AB2008/10)<br>*Chlamydomonas reinhardtii* (SAG 11-32b)*<br>*Chlorella vulgaris* (SAG 211-11b)<br>*Cryptomonas sp.* (26-80)*<br>*Cyclotella meneghiniana* (SAG 1020-1a)<br>*Monoraphidium minutum* (243-1)*<br>*Navicula pelliculosa* (SAG 1050-3)<br>*Oocystis marsonii* (SAG 257-1)<br>*Pandorina morum* (SAG 2017)	*Resident Community B*

Supplementary Table 2 Mean cell size (µm^3/ species with *: width in µm), maximum gross growth rates (and edibility for *Brachionus calyciflorus* (Bc) and *Cephalodella sp.* (Cs) of the resident species of the experiments. Data are from all experiments.

	**Size - start**	**growth rates**	**edibility**
Acutodesmus obliquus (SAG 276-3a)	67	high	Edible by Bc
Chlamydomonas reinhardtii (SAG 11-32b)	235	high	Edible by Bc and Cs
Cryptomonas sp. (26-80)	410	high	Edible by Bc and Cs
Monoraphidium minutum (243-1)	31	high	Edible by Bc and Cs
Peridinium sp. (SAG 2017)	31381	low	Inedible for BC and CS
Planktothrix aghardii (NIVA-CYA 34)*	5.8	low	Edible by Bc
Stephanodiscus hantzschii (SAG 1020-1a)	61	low	Edible by Bc and Cs
Synechococcus elongates (SAG 89.79)	0.5	high	Edible by Bc and Cs
Chlorella vulgaris (SAG 211-11b)*	10.6	high	Edible by Bc and Cs
Aphanizomenon gracile (AB2008/10)*	4.4	low	Hardly edible for Bc Inedible for Cs
Cyclotella meneghiniana (SAG 1020-1a)	5.3	low	Edible by Bc and Cs
Navicula pelliculosa (SAG 1050-3)	40	high	Edible by Bc
Oocystis marsonii (SAG 257-1)	41.2	high	Edible by Bc
Pandorina morum (SAG 2017)	14225	high	Inedible for BC and CS

C. raciborskii	Single strains										3 strains														
19F6	X										X			X			X				X		X		
26D9		X									X	X							X			X			X
27F11*			X										X		X		X							X	
AB2008/71*				X							X						X			X			X	X	
MEL07					X							X		X				X							X
Peter149						X									X	X				X	X				X
Peter163							X							X				X	X			X			
SP08-4								X					X		X				X				X		
ZIE05									X			X				X		X				X		X	
ZIE11										X			X			X				X	X				

C. raciborskii	6 Strains															All 10 strains		
19F6	X	X	X	X		X	X				X	X	X			X	X	X
26D9	X				X	X	X	X	X		X					X	X	X
27F11*		X	X	X		X			X	X	X		X	X	X	X	X	X
AB2008/71*	X			X	X		X	X	X		X	X		X		X	X	X
MEL07	X		X	X	X		X		X	X		X		X	X	X	X	X
Peter149		X		X				X		X	X	X		X	X	X	X	X
Peter163		X	X		X	X		X	X	X			X	X	X	X	X	X
SP08-4	X	X	X		X	X		X	X			X	X			X	X	X
ZIE05		X		X			X	X		X	X		X	X	X	X	X	X
ZIE11	X		X		X	X	X			X		X	X		X	X	X	X

* not in exp. IV, additional strain 22F8

Supplementary Figure 1 Composition of the *Raphidiopsis raciborskii* populations in the experiments.

Primer Design

Sequencing and generating of assemblies

Libraries of fragment sizes between ~ 430 and 620 bp were prepared from the genomic DNA of 12 strains of *Raphidiopsis raciborskii* (see Bolius et al., 2017) – 10 strains used in the given experiment) and sequenced on an Illumina NextSeq to generate 150 bp paired-end reads. Between 0.4 and 0.8 Gbp of raw data were generated for each of the strains. SeqPrep (John, 2011) was used for adapter trimming and quality filtering (q 20). Genomes were then assembled using miraculous (Chapman et al., 2011); k-mer size and minimum k-mer cutoff were set to auto-detect. Assemblies for strain 26D9 never completed, regardless of parameter settings. k-mer plots generated using kmergenie (Chikhi and Medvedev, 2014) suggested a large amount of contamination in this sample as a possible reason for its failure to assemble. All other strains were assembled into 240 to 1,770 scaffolds (median: 309), totalling between 3.5 and 13.4 Mbp (median: 7.5). N50 values ranged between 7.6 and 200.5 kbp (median: 57.1).

Identification of R. raciborskii scaffolds

As the genome size of *C. raciborskii* is expected to be ~ 4 Mbp, the assembly results indicated contamination present in the data. To exclude contaminating scaffolds, all assemblies were compared against three publicly available *R. raciborskii* genomes. These reference data consisted of 93 scaffolds from strain CS-505 (GenBank Accession ACYA00000000), and 1,510 and 924 scaffolds of at least 200 bp from strains CS-506 (SRR1042336) and CS-509 (SRR1041118), respectively (downloaded from JGI-IMG in November 2015). Only scaffolds from our assemblies that aligned to any of these references using MUMmer (Kurtz et al., 2004) were used for further analysis; they made up a total of 2.9 to 3.9 Mbp for each of our strains.

Prediction of PCR primers

To identify genomic regions that were available for all nine assemblies, we used the software MUGSY (Angiuoli and Salzberg, 2011) to generate multiple alignments from the scaffolds. Only alignment regions with data for all nine strains were used for further analysis. Alignment regions with SNPs or indels that were diagnostic for individual strains or groups of strains were identified using custom Perl scripts that made use of BioPerl (Stajich et al., 2002). From these alignment regions, PCR

primers were predicted using a local installation of Primer3 (Koressaar and Remm, 2007; Untergasser et al., 2012). BLAST (Altschul et al., 1990) was used to compare all candidate primers with Peter09.1, which contained the most assembled data after excluding contamination. Candidate primers that matched more than one genomic region of Peter09.1 were excluded from further consideration.

For this study, nine primer pairs were manually selected (Table S2). Five of these primer pairs can unambiguously identify individual strains based on SNPs (24F11, AB2008/71, Peter07_163 and SP08-4) or based on SNPs and length polymorphism (19F6). The other strains, however, cannot be distinguished unambiguously: PrimerPair MEL07 has identical sequences and lengths for MEL07 and Peter07_149. The amplified product from PrimerPair Peter07_149 generates sequences that are identical in length and sequence between 19F6, AB2008/71, MEL07, and Peter07_149. PrimerPair ZIE05 generates products of the same lengths for all nine strains but generates three different fragments that slightly differ in sequence (ZIE05 & ZIE11 vs. SP08-4 vs. the rest). Finally, PrimerPair ZIE11 generates fragments of equal lengths for all strains but can not distinguish between AB2008/71, SP08-4, ZIE05, and ZIE11 due to shared variation between these four strains in the amplified region.

References:

Altschul, S. F., Gish, W., Miller, W., Myers, E. W., and Lipman, D. J. (1990). Basic local alignment search tool. *J. Mol. Biol.* 215, 403–410. doi:10.1016/S0022-2836(05)80360-2.

Angiuoli, S. V., and Salzberg, S. L. (2011). Mugsy: Fast multiple alignment of closely related whole genomes. *Bioinformatics* 27, 334–342. doi:10.1093/bioinformatics/btq665.

Bolius, S., Wiedner, C., and Weithoff, G. (2017). High local trait variability in a globally invasive cyanobacterium. *Freshw. Biol.* 62, 1879–1890. doi:10.1111/fwb.13028.

Chapman, J. A., Ho, I., Sunkara, S., Luo, S., Schroth, G. P., and Rokhsar, D. S. (2011). Meraculous: De novo genome assembly with short paired-end reads. *PLoS One* 6. doi:10.1371/journal.pone.0023501.

Chikhi, R., and Medvedev, P. (2014). Informed and automated k-mer size selection for genome assembly. *Bioinformatics* 30, 31–37. doi:10.1093/bioinformatics/btt310.

John, J. (2011). SeqPrep: Tool for stripping adaptors and/or merging paired reads with overlap into single reads. *https://githubcom/jstjohn/SeqPrep*.

Koressaar, T., and Remm, M. (2007). Enhancements and modifications of primer design program Primer3. *Bioinformatics* 23, 1289–1291. doi:10.1093/bioinformatics/btm091.

Kurtz, S., Phillippy, A., Delcher, A. L., Smoot, M., Shumway, M., Antonescu, C., et al. (2004). Versatile and open software for comparing large genomes. *Genome Biol.* 5, R12. doi:10.1186/gb-2004-5-2-r12.

Stajich, J. E., Block, D., Boulez, K., Brenner, S. E., Chervitz, S. A., Dagdigian, C., et al. (2002). The Bioperl toolkit: Perl modules for the life sciences. *Genome Res.* 12, 1611–8. doi:10.1101/gr.361602.

Untergasser, A., Cutcutache, I., Koressaar, T., Ye, J., Faircloth, B. C., Remm, M., et al. (2012). Primer3-new capabilities and interfaces. *Nucleic Acids Res.* 40, 1–12. doi:10.1093/nar/gks596.

Supplementary Table 3 Specific primer pairs for the strains of *Raphidiopsis raciborskii.*

strain	forward	reverse	Sequences
19F6	CGTTTAGATGCTTGCTGCCA	TCCAAGATTACAGCTCCCGA	MT531416 - 26
27F11	AAGTCACCCGGGGGAGTG	GGTTTTCCAGTGGGCATGG	MT531438 - 48
AB2008/71	AGTTGGCTCAATTCCTGTGGA	TGAAGCAGGAGCAAAACCAAG	MT531449 - 59
MEL07	ACGATCAACACTCAAAGCGA	GCACTTTCAGATCCCCAAATGG	MT531460 - 70
Peter07_149	CTCCGATGGGATTATGGCAGT	AGCTGAGGAAGTACCGCT	MT531471 - 81
Peter07_163	AGCGCGGTTGATTACATCC	AGCACCATGGCATTCAACCT	MT531482 - 92
SP08-4	GCCCGTGTGGTGCTAGAC	AGCTTTGGCCAAACTCGA	MT531504 - 14
ZIE05	TAGTGCGATCGCCAGACT	GTTTGTTCTTTCAGGTCAAGCA	MT531515 - 25
ZIE11	TCCTGACAGCAAGGGGGA	GAGAGAAACATCCAACTCTGCA	MT531526 - 36

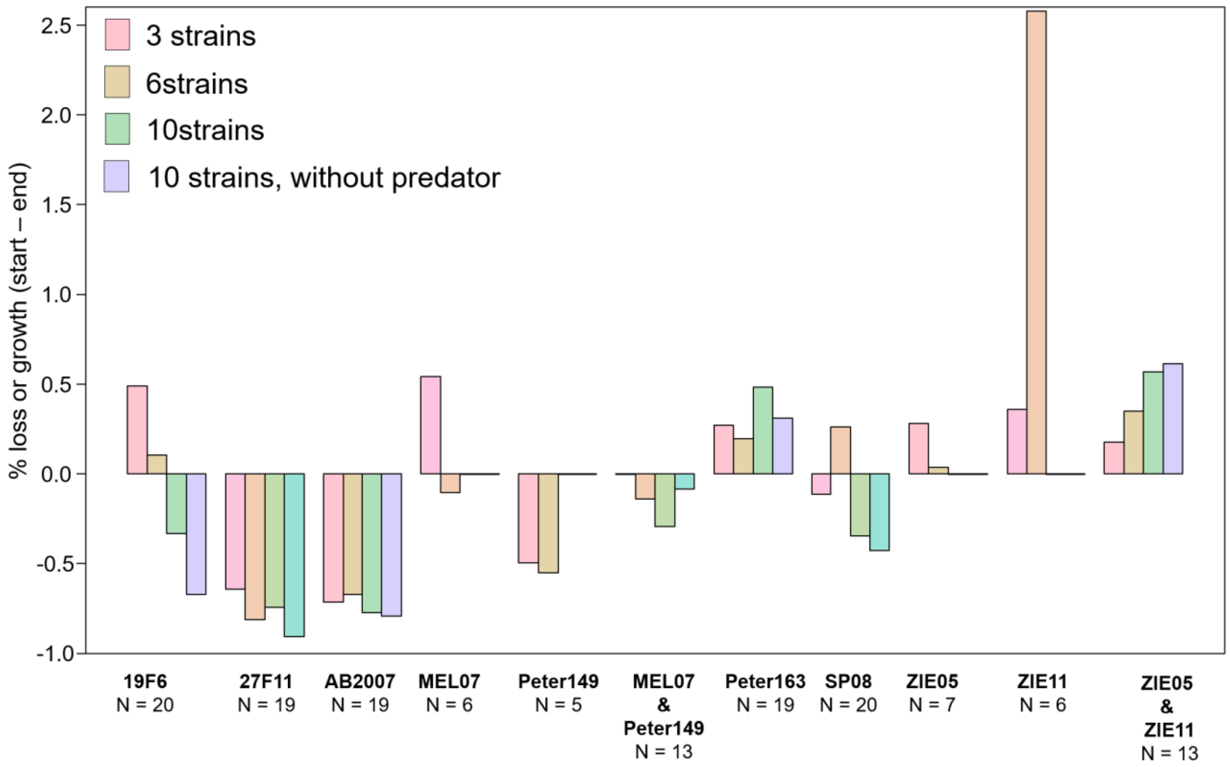

Supplementary Figure 2 Loss and gain (in percentage) of the *Raphidiopsis raciborskii* strains in the mixtures from the invasion event to the end of the experiment **III**. It is plotted by the strains and considering the level the level of strain diversity (3, 6, 10, 10 without rotifers).

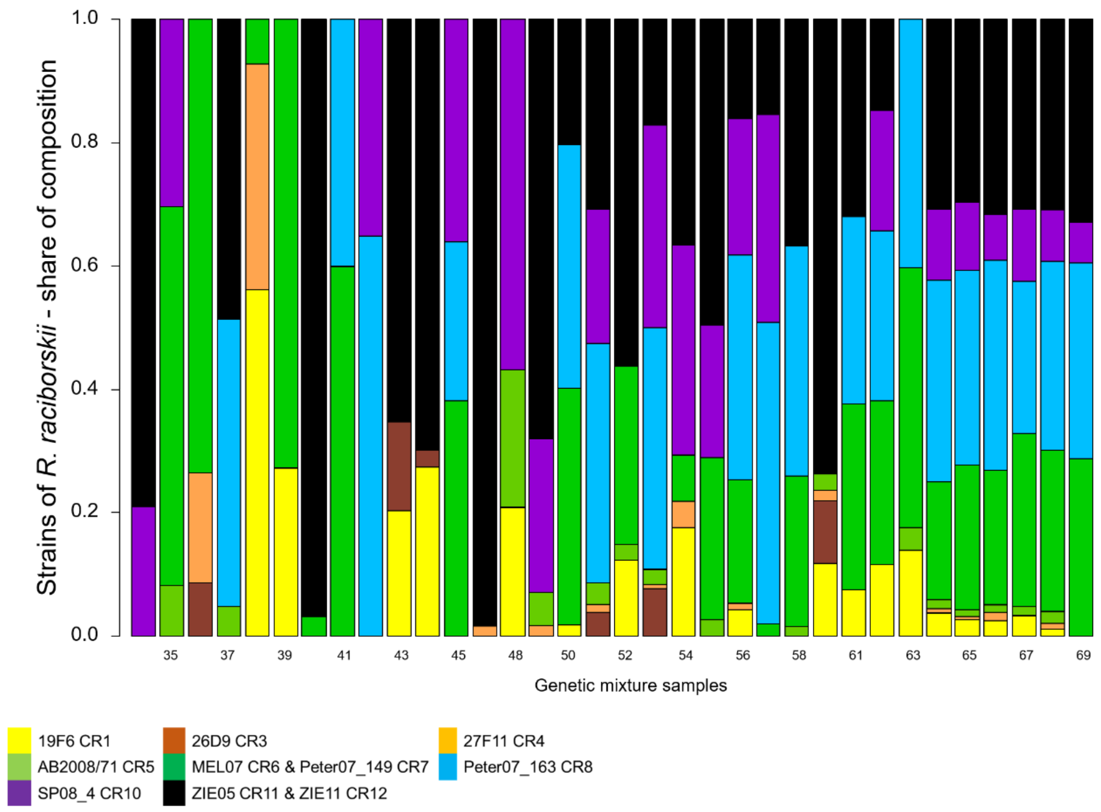

Supplementary Figure 3 Sstrain composition of *Raphidiopsis raciborskii* at the end of experiment **III**.

Supplementary Table 4 Nitrogen (N) –gain of all strains in experiment **III** on day 38 measured as surplus in particulate N on top of nitrogen in the medium (51 µmol).

strain	N-fixation
ZIE 05	95.43
SP08-4	92.25
Peter07-149	27.97
ZIE 11	137.49
AB2008-7	50.19
19F6	35.90
Peter07-163	96.22
27F11	50.98
MEL07	77.97
26D9	31.94
